# Supplementary material for: The Systems Biology Research Tool: evolvable open-source software
Source: BMC Syst Biol. 2008 Jun 29;2:55. doi: 10.1186/1752-0509-2-55 (PMC2446383; doi:10.1186/1752-0509-2-55)
Supplement: Additional file 1 — SBRT Archive. An archive of the current version of the Systems Biology Research Tool. [file 1752-0509-2-55-S1.zip › sbrt-1.4.0/doc/developers_guide/api/sbrt/kernel/math/optimization/package-summary.html]

sbrt.kernel.math.optimization


|  |  |  |
| --- | --- | --- |
| **Overview** \| **Package** \| Class \| **Use** \| **Tree** \| **Deprecated** \| **Index** \| **Help** | |  |
| **PREV PACKAGE**   **NEXT PACKAGE** | **FRAMES**    **NO FRAMES**     **All Classes** |  |


---

## Package sbrt.kernel.math.optimization

Provides classes and interfaces for creating objects relevant to solving
optimization problems.

**See:**
  
          **Description**

| **Interface Summary** | |
| --- | --- |
| **LinearProgramSolver<R,C>** | This interface is used to represent linear program solvers. |
| **ProgramSolver<R,C>** | This interface is used to represent program solvers, as in linear or quadratic program solvers. |
| **QuadraticProgramSolver<R,C>** | This interface is used to represent quadratic program solvers. |

| **Enum Summary** | |
| --- | --- |
| **OptimizationSense** | This enum type is used to represent the sense, or direction, of an optimization problem. |
| **ProgramSolverStatus** | This enum type is used to indicate the status of a program solver. |

| **Exception Summary** | |
| --- | --- |
| **ProgramSolverException** | This class is used to wrap exceptions thrown by program solver packages. |

## Package sbrt.kernel.math.optimization Description

Provides classes and interfaces for creating objects relevant to solving
optimization problems.

---


|  |  |  |
| --- | --- | --- |
| **Overview** \| **Package** \| Class \| **Use** \| **Tree** \| **Deprecated** \| **Index** \| **Help** | |  |
| **PREV PACKAGE**   **NEXT PACKAGE** | **FRAMES**    **NO FRAMES**     **All Classes** |  |


---
